# Supplementary material for: Efficacy of Household and Commercial Washing Agents in Removing the Pesticide Thiabendazole Residues from Fruits
Source: Foods. 2025 Jan 18;14(2):318. doi: 10.3390/foods14020318 (PMC11764615; doi:10.3390/foods14020318)
Supplement: Supplementary file 1 [file foods-14-00318-s001.zip › foods-3410785-supplementary.pdf]

Table S1 Comparison of different Fruit & Vegetable Wash products and their instruction.

Product Name	Price	Suggested commercial instruction for removing residues on apple
Product 1	$21.99/48oz	Spray and stand 15 min, and then rinse with water for 30 sec
Product 2	$29.99/64oz	Submerge using 3% product dilution for 3 min, and then rinse with water for 30 sec
Product 3	$21.95/64oz	Submerge using 2% product dilution for 5 min, and then rinse with water for 30 sec
Product 4	$23.99/64oz	Spray and stand 15 min, and then rinse with water for 30 sec

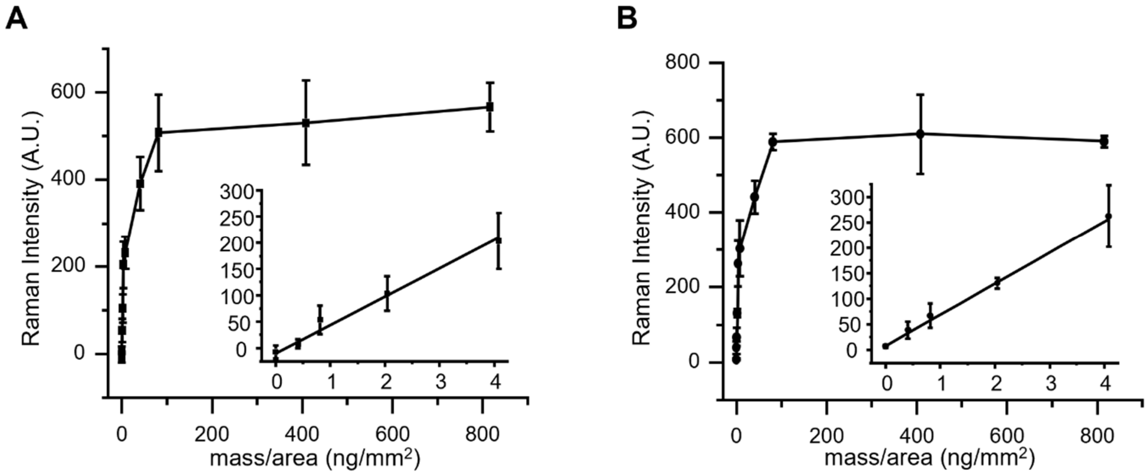

Figure S1 Plot of peak height at 1010 cm^{-1} vs. pesticide amount per unit area (mass/area) and the corresponding linear fitting curve on apple treated with (A) T (thiabendazole alone) and (B) TA (thiabendazole with Alligare 90®).

Table S2 Raman peak heights, estimated residue values and corresponding reduction (%) of thiabendazole on apple treated with 1000ppm thiabendazole solution following 10 min soaking treatment using different suspensions (n = 4).

Washing agent	Starch Concentration	Raman peak intensity (a.u.) (mean±SD)	Residue value (ng/mm^2) (mean±SD)	Reduction (%)
All-purpose flour	2%	370.45 ± 34.39 bc	35.98 ± 8.94 bc	95.59
Corn starch	1%	441.77 ± 25.60 b	56.83 ± 8.84 a	93.04
	2%	322.43 ± 30.63 c	24.69 ± 6.58 c	96.98
Rice flour	2%	413.57 ± 5.91 b	47.56 ± 1.77 a	94.17
Water	0%	517.93 ± 37.68 a	NA	< 90

Values followed by different letters are significantly different at α = 0.05.

Table S3 Raman peak heights, estimated residue values and corresponding reduction (%) of thiabendazole on apple treated with 100ppm thiabendazole solution following 5 min soaking treatment using different solutions (n = 4).

Soaking solution	Raman peak intensity (a.u.) (mean±SD)	Residue value (ng/mm^2) (mean±SD)	Reduction (%)
2% corn starch	320.57 ± 29.17	24.27 ± 6.10 b	70.27
5% baking soda	303.60 ± 42.76	21.10 ± 8.36 b	74.15
Mixture	397.02 ± 36.80	43.25 ± 9.92 a	47.01

Values followed by different letters are significantly different at α = 0.05.

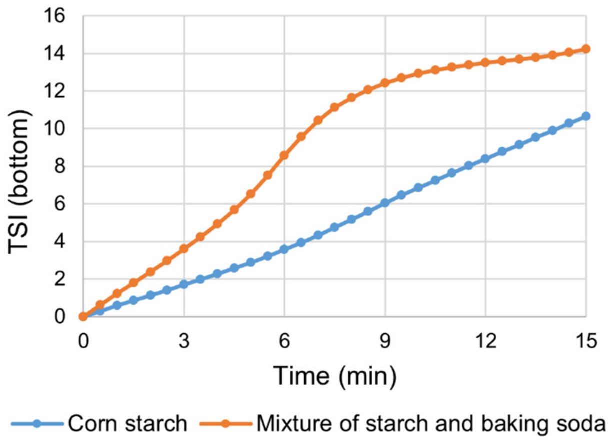

Figure S2 The change of TSI of the bottom layer of pure corn starch suspension and mixture of corn starch and baking soda over storage time.

Table S4 Raman peak heights, estimated residue values and corresponding reduction (%) of thiabendazole on apple treated with T (thiabendazole alone) and TA (thiabendazole with Alligare 90®) following 10 min homemade soaking treatment (n = 4).

Soaking strategy	Pesticide formula	Raman peak intensity (a.u.) (mean±SD)	Residue value (ng/mm^2) (mean±SD)	Reduction (%)
2% corn starch	T	387.40 ± 40.34	24.96 ± 4.54 a	69.42
	TA	386.95 ± 40.55	27.43 ± 9.57 a	66.39
5% baking soda	T	281.88 ± 34.72	16.76 ± 6.65 abc	79.46
	TA	338.87 ± 21.79	16.10 ± 4.97 abc	80.27
2% corn starch followed by 5% baking soda	T	180.75 ± 58.01	4.79 ± 2.47 c	94.13
	TA	267.29 ± 50.86	6.71 ± 2.25 bc	91.78
5% baking soda followed by 2% corn starch	T	304.61 ± 17.86	20.83 ± 3.47 ab	74.48
	TA	334.92 ± 5.40	15.17 ± 1.22 abc	81.42

Values followed by different letters are significantly different at α = 0.05.

Table S5 Raman peak heights, estimated residue values and corresponding reduction (%) of thiabendazole on apple treated with T (thiabendazole alone) and TA (thiabendazole with Alligare 90®) after treatment following the instructions of varied commercial wash products (n = 4).

Washing agent	Pesticide formula	Raman peak intensity (a.u.) (mean±SD)	Residue value (ng/mm^2) (mean±SD)	Removal efficiency (%)
Commercial instruction 1	T	387.40 ± 40.34	40.76 ± 10.55 a	50.07
	TA	407.71 ± 51.67	32.58 ± 12.50 a	60.09
Commercial instruction 2	T	328.77 ± 23.29	25.94 ± 5.22 a	68.22
	TA	370.64 ± 16.20	23.41 ± 3.79 a	71.31
Commercial instruction 3	T	356.01 ± 42.50	33.28 ± 7.54 a	59.23
	TA	370.79 ± 27.62	23.50 ± 6.52 a	71.21
Commercial instruction 4	T	337.47 ± 73.17	29.43 ± 16.63 a	63.95
	TA	365.94 ± 42.70	22.48 ± 10.14 a	72.46

Values followed by different letters are significantly different at α = 0.05.

Table S6 Raman peak heights, estimated residue values and corresponding reduction (%) of thiabendazole on apple treated with T (thiabendazole alone) and TA (thiabendazole with Alligare 90®) after 5 min soaking treatment using the 2% dilution of varied commercial wash products (n = 4).

Soaking agent	Pesticide formula	Raman peak intensity (a.u.) (mean±SD)	Residue value (ng/mm^2) (mean±SD)	Removal efficiency (%)
Commercial soaking 1	T	303.31 ± 44.88	21.11 ± 8.57 ab	74.13
	TA	351.57 ± 53.13	19.24 ± 12.54 ab	76.42
Commercial soaking 2	T	293.48 ± 6.03	18.60 ± 1.16 ab	77.21
	TA	339.74 ± 11.57	16.26 ± 2.63 ab	80.07
Commercial soaking 3	T	366.20 ± 42.50	33.28 ± 7.54 a	59.23
	TA	370.79 ± 27.62	23.50 ± 6.52 ab	71.21
Commercial soaking 4	T	169.37 ± 52.01	3.84 ± 1.73 b	95.30
	TA	222.94 ± 11.47	3.52 ± 0.19 b	95.69

Values followed by different letters are significantly different at α = 0.05.

Table S7 Comparison of chemical ingredients of various fresh produce wash products.

	Product 1	Product 2	Product 3	Product 4
pH control agent	Sodium Bicarbonate [a]		Sodium Hydroxide [ab]	
Non-ionic surfactant	Lauryl Glucoside; Caprylyl/capryl Oligoglucoside		Decyl Glucoside [b]	Decyl Glucoside [b]; Lauryl glucoside
Emulsifier				Oleic acid [ab]; Potassium Oleate [a]
Natural solvent	Lemon Oil [a]	Denatured Lemon Oil [a]; Sunflower Oil [a]; Grapefruit Oil [a]	Orange Peel Extract [a]	D-limonene [a]; Orange Peel Oil [a]; Sunflower Seed Oil [a]
Chelating agent			Lactic Acid [a]	
Abrasive agent	Sodium Chloride [a]			
Humectant		Glycerin [a]	Glycerin [a]	Glycerin [a]; Rosemary Leaf Extract [a]
Saponifying agent		Potassium Hydroxide [a]		Potassium Hydroxide [a]
Preservative	Benzisothiazolinone [a]			Potassium Sorbate [a]

a. Substances approved for use in Food according to FDA regulations (21 CFR)
b. Inert ingredients approved for use in pre- and post-harvest (exemptions from the requirement of a tolerance) according to FDA regulations (40 CFR § 180.910)
